# *N*-(2-Methyl­phen­yl)-6-(1*H*-pyrazol-1-yl)pyridazin-3-amine

**DOI:** 10.1107/S1600536809022867

**Published:** 2009-06-20

**Authors:** Abdul Qayyum Ather, M. Nawaz Tahir, Misbahul Ain Khan, Muhammad Makshoof Athar

**Affiliations:** a Department of Chemistry, Islamia University, Bahawalpur, Pakistan, and, Applied Chemistry Research Center, PCSIR Laboratories complex, Lahore 54600, Pakistan; b Department of Physics, University of Sargodha, Sargodha, Pakistan; c Department of Chemistry, Islamia University, Bahawalpur, Pakistan; d Institute of Chemistry, University of the Punjab, Lahore, Pakistan

## Abstract

The title compound, C_14_H_13_N_5_, crystallizes with two crystallographically independent mol­ecules in the unit cell. The two mol­ecules form dimers through inter­molecular N—H⋯N and C—H⋯N hydrogen bonds. The hydrogen-bonding motifs are *R*_2_^2^(8) for both the N—H⋯N and C—H⋯N inter­actions. The pyrazole and pyrimidine rings form dihedral angles of 6.2 (3) and 8.3 (3)° with each other and the dihedral angles between the pyrazole and benzene rings are 54.9 (2) and 58.6 (2)°. The benzene rings of neighbouring dimers also exhibit C—H⋯π inter­actions.

## Related literature

A docking study of pyrazololylpyridazine has shown inhibitory action against glycogen synthase kinase 3, see: Xiao *et al.* (2006 ▶); For graph-set notation, see: Bernstein *et al.* (1995 ▶).
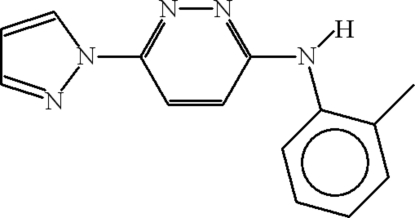


## Experimental

### 

#### Crystal data

C_14_H_13_N_5_
*M* *_r_* = 251.29
Monoclinic, 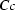

*a* = 16.548 (5) Å
*b* = 19.639 (4) Å
*c* = 8.015 (5) Å
β = 99.619 (5)°
*V* = 2568.1 (19) Å^3^
*Z* = 8
Mo *K*α radiation
μ = 0.08 mm^−1^
*T* = 296 K
0.24 × 0.20 × 0.18 mm

#### Data collection

Bruker Kappa APEXII CCD diffractometer
Absorption correction: multi-scan (*SADABS*; Bruker, 2005 ▶) *T*_min_ = 0.982, *T*_max_ = 0.988
13833 measured reflections
3209 independent reflections
1716 reflections with *I* > 2σ(*I*)
*R*_int_ = 0.042

#### Refinement

*R*[*F* ^2^ > 2σ(*F* ^2^)] = 0.063
*wR*(*F* ^2^) = 0.167
*S* = 1.02
3209 reflections
303 parameters
2 restraints
H-atom parameters constrained
Δρ_max_ = 0.40 e Å^−3^
Δρ_min_ = −0.32 e Å^−3^

### 

Data collection: *APEX2* (Bruker, 2007 ▶); cell refinement: *SAINT* (Bruker, 2007 ▶); data reduction: *SAINT*; program(s) used to solve structure: *SHELXS97* (Sheldrick, 2008 ▶); program(s) used to refine structure: *SHELXL97* (Sheldrick, 2008 ▶); molecular graphics: *ORTEP-3 for Windows* (Farrugia, 1997 ▶) and *PLATON* (Spek, 2009 ▶); software used to prepare material for publication: *WinGX* (Farrugia, 1999 ▶) and *PLATON*.

## Supplementary Material

Crystal structure: contains datablocks global, I. DOI: 10.1107/S1600536809022867/zl2217sup1.cif

Structure factors: contains datablocks I. DOI: 10.1107/S1600536809022867/zl2217Isup2.hkl

Additional supplementary materials:  crystallographic information; 3D view; checkCIF report

## Figures and Tables

**Table 1:** Hydrogen-bond geometry (Å, °)

*D*—H⋯*A*	*D*—H	H⋯*A*	*D*⋯*A*	*D*—H⋯*A*
N1—H1⋯N7^i^	0.86	2.26	3.086 (5)	162
N6—H6*A*⋯N2^ii^	0.86	2.26	3.065 (6)	156
C7—H7*C*⋯N8^i^	0.96	2.53	3.482 (8)	175
C21—H21*C*⋯N3^ii^	0.96	2.59	3.519 (10)	163
C6—H6⋯*Cg*1	0.93	2.80	3.529 (6)	136

Symmetry codes: (i) 
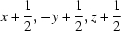
; (ii) 
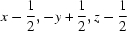
. *Cg*1 is the centroid of benzene ring (C15–C20).

**Fig. 1.**
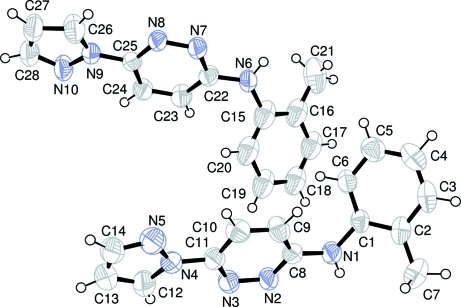
View of the title compound with the atom numbering scheme. The thermal ellipsoids are drawn at the 50% probability level. H-atoms are shown as small spheres of arbitrary radii.

**Fig. 2.**
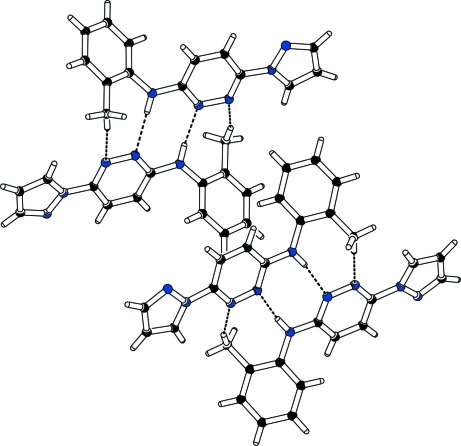
Packing diagram of the title compound (PLATON: Spek, 2009) showing the dimers and ring motifs. Hydrogen bonds are symbolized by dashed lines.

## supplementary crystallographic information

### Comment

Pyrazolylpyridazine derivatives are potential anticholestromic and antihypertensive agents. A docking study of pyrazololylpyridazine has shown inhibitory action against glycogen synthase kinase 3 (Xiao *et al.*, 2006). In continuation of our work on the synthesis and reactions of azolylpyridazines, we have isolated crystals of the title compound, (Fig. 1).

The title compound contains pyrazole, pyridazine and benzene rings. In the asymmetric unit there are two molecules which differ from one another crystallographically.

In one molecule (containing C1—C14), the pyrazole ring is oriented at dihedral angles of 6.16 (30)° and 54.91 (21)° with the pyridazine and benzene rings, respectively. In the second molecule, the pyrazole ring exhibits dihedral angles of 8.26 (34)° and 58.57 (20)° with the pyridazine and benzene rings, respectively. Through intermolecular N–H···N and C–H···N hydrogen bonds the two molecules form dimers with hydrogen bonding ring motifs of *R*_2_^2^(8) (Bernstein *et al.*, 1995). C—H···π interactions between the benzene rings are also observed in the structure of the title compound (Table 1, *Cg*1 is the centroid of benzene ring (C15—C20)).

### Experimental

3-Chloro-6-(1*H*-pyrazol-1-yl)pyridazine (1.68 g, 9.33 mmol) and 2-toluidine (1 g, 9.34 mmol) were refluxed in dimethylformamide (DMF) for 2 h. The reaction mixture was concentrated under vacuum and poured in cold water. The precipitates obtained were filtered, washed with distilled water and dried to give 51.28% yield. The product obtained was purified by column chromatography and recrystallized in benzene.

### Refinement

In the absence of significant anomalous scattering effects, Friedel pairs were merged. The atoms of one of the benzene rings were refined with equal anisotropic thermal parameters.

H-atoms were positioned geometrically, with N—H = 0.86 Å, C—H = 0.93 and 0.96 Å for aromatic rings and metyl H-atoms and constrained to ride on their parent atoms, with U_iso_(H) = xU_eq_(C, N), where x = 1.5 for methyl H and x = 1.2 for all other H atoms.

### Crystal data

**Table:**

C_14_H_13_N_5_	*F*(000) = 1056
*M**_r_* = 251.29	*D*_x_ = 1.300 Mg m^−^^3^
Monoclinic, *C**c*	Mo *K*α radiation, λ = 0.71073 Å
Hall symbol: C -2yc	Cell parameters from 2920 reflections
*a* = 16.548 (5) Å	θ = 2.1–28.4°
*b* = 19.639 (4) Å	µ = 0.08 mm^−^^1^
*c* = 8.015 (5) Å	*T* = 296 K
β = 99.619 (5)°	Prismatic, white
*V* = 2568.1 (19) Å^3^	0.24 × 0.20 × 0.18 mm
*Z* = 8	

### Data collection

**Table:**

Bruker Kappa APEXII CCD diffractometer	3209 independent reflections
Radiation source: fine-focus sealed tube	1716 reflections with *I* > 2σ(*I*)
graphite	*R*_int_ = 0.042
Detector resolution: 7.40 pixels mm^-1^	θ_max_ = 28.4°, θ_min_ = 2.1°
ω scans	*h* = −22→22
Absorption correction: multi-scan (*SADABS*; Bruker, 2005)	*k* = −24→26
*T*_min_ = 0.982, *T*_max_ = 0.988	*l* = −8→10
13833 measured reflections	

### Refinement

**Table:**

Refinement on *F*^2^	Primary atom site location: structure-invariant direct methods
Least-squares matrix: full	Secondary atom site location: difference Fourier map
*R*[*F*^2^ > 2σ(*F*^2^)] = 0.063	Hydrogen site location: inferred from neighbouring sites
*wR*(*F*^2^) = 0.167	H-atom parameters constrained
*S* = 1.01	*w* = 1/[σ^2^(*F*_o_^2^) + (0.056*P*)^2^ + 2.2381*P*] where *P* = (*F*_o_^2^ + 2*F*_c_^2^)/3
3209 reflections	(Δ/σ)_max_ < 0.001
303 parameters	Δρ_max_ = 0.40 e Å^−^^3^
2 restraints	Δρ_min_ = −0.32 e Å^−^^3^

### Special details

**Table:**

Geometry. All e.s.d.'s (except the e.s.d. in the dihedral angle between two l.s. planes) are estimated using the full covariance matrix. The cell e.s.d.'s are taken into account individually in the estimation of e.s.d.'s in distances, angles and torsion angles; correlations between e.s.d.'s in cell parameters are only used when they are defined by crystal symmetry. An approximate (isotropic) treatment of cell e.s.d.'s is used for estimating e.s.d.'s involving l.s. planes.
Refinement. Refinement of *F*^2^ against ALL reflections. The weighted *R*-factor *wR* and goodness of fit *S* are based on *F*^2^, conventional *R*-factors *R* are based on *F*, with *F* set to zero for negative *F*^2^. The threshold expression of *F*^2^ > σ(*F*^2^) is used only for calculating *R*-factors(gt) *etc*. and is not relevant to the choice of reflections for refinement. *R*-factors based on *F*^2^ are statistically about twice as large as those based on *F*, and *R*- factors based on ALL data will be even larger.

### Fractional atomic coordinates and isotropic or equivalent isotropic displacement parameters (Å^2^)

**Table:**

	*x*	*y*	*z*	*U*_iso_*/*U*_eq_	
N1	0.3430 (2)	0.3434 (2)	0.7950 (6)	0.0617 (12)	
H1	0.3887	0.3649	0.8034	0.074*	
N2	0.4170 (2)	0.2448 (2)	0.8037 (6)	0.0629 (12)	
N3	0.4249 (2)	0.1762 (2)	0.8272 (6)	0.0640 (12)	
N4	0.3754 (3)	0.0702 (2)	0.8745 (6)	0.0615 (12)	
N5	0.3154 (3)	0.0302 (3)	0.9209 (8)	0.0904 (18)	
N6	0.0751 (3)	0.1921 (2)	0.2213 (6)	0.0609 (12)	
H6A	0.0288	0.2129	0.2121	0.073*	
N7	0.0075 (2)	0.0924 (2)	0.2451 (5)	0.0562 (11)	
N8	0.0015 (2)	0.0239 (2)	0.2363 (6)	0.0544 (10)	
N9	0.0521 (2)	−0.08308 (18)	0.1990 (5)	0.0522 (10)	
N10	0.1036 (3)	−0.1245 (2)	0.1338 (6)	0.0680 (13)	
C1	0.2696 (3)	0.3825 (2)	0.7595 (6)	0.0499 (11)	
C2	0.2655 (3)	0.4452 (2)	0.8362 (6)	0.0567 (13)	
C3	0.1903 (4)	0.4801 (3)	0.7933 (8)	0.0714 (16)	
H3	0.1850	0.5229	0.8401	0.086*	
C4	0.1249 (4)	0.4537 (3)	0.6860 (9)	0.0779 (17)	
H4	0.0762	0.4781	0.6631	0.093*	
C5	0.1306 (3)	0.3920 (3)	0.6125 (8)	0.0714 (15)	
H5	0.0863	0.3739	0.5390	0.086*	
C6	0.2032 (3)	0.3570 (3)	0.6494 (7)	0.0556 (12)	
H6	0.2078	0.3149	0.5988	0.067*	
C7	0.3362 (4)	0.4739 (3)	0.9540 (8)	0.0779 (18)	
H7A	0.3461	0.4469	1.0552	0.117*	
H7B	0.3241	0.5199	0.9823	0.117*	
H7C	0.3840	0.4735	0.9008	0.117*	
C8	0.3467 (3)	0.2744 (2)	0.8169 (6)	0.0521 (12)	
C9	0.2804 (3)	0.2371 (3)	0.8593 (7)	0.0613 (14)	
H9	0.2325	0.2590	0.8752	0.074*	
C10	0.2878 (3)	0.1693 (3)	0.8763 (7)	0.0599 (14)	
H10	0.2443	0.1425	0.8979	0.072*	
C11	0.3625 (3)	0.1409 (3)	0.8603 (7)	0.0556 (12)	
C12	0.4397 (4)	0.0335 (3)	0.8451 (9)	0.0790 (17)	
H12	0.4871	0.0504	0.8118	0.095*	
C13	0.4230 (5)	−0.0330 (3)	0.8729 (11)	0.098 (2)	
H13	0.4560	−0.0707	0.8636	0.117*	
C14	0.3461 (5)	−0.0318 (4)	0.9180 (11)	0.105 (3)	
H14	0.3187	−0.0707	0.9437	0.126*	
C15	0.1471 (2)	0.23170 (19)	0.2472 (5)	0.0810 (8)	
C16	0.1493 (2)	0.2952 (2)	0.1713 (5)	0.0810 (8)	
C17	0.2214 (2)	0.33286 (16)	0.1961 (5)	0.0810 (8)	
H17	0.2229	0.3753	0.1453	0.097*	
C18	0.2913 (2)	0.30707 (19)	0.2966 (6)	0.0810 (8)	
H18	0.3395	0.3323	0.3131	0.097*	
C19	0.2891 (2)	0.2436 (2)	0.3724 (5)	0.0810 (8)	
H19	0.3358	0.2263	0.4397	0.097*	
C20	0.2170 (2)	0.20591 (16)	0.3477 (5)	0.0810 (8)	
H20	0.2155	0.1634	0.3984	0.097*	
C21	0.0748 (5)	0.3242 (3)	0.0633 (10)	0.104 (3)	
H21A	0.0527	0.2915	−0.0212	0.156*	
H21B	0.0894	0.3649	0.0093	0.156*	
H21C	0.0344	0.3347	0.1326	0.156*	
C22	0.0745 (3)	0.1232 (2)	0.2100 (6)	0.0515 (12)	
C23	0.1379 (3)	0.0856 (3)	0.1572 (7)	0.0599 (13)	
H23	0.1829	0.1076	0.1264	0.072*	
C24	0.1322 (3)	0.0166 (3)	0.1517 (6)	0.0551 (13)	
H24	0.1734	−0.0102	0.1202	0.066*	
C25	0.0619 (3)	−0.0118 (2)	0.1954 (6)	0.0466 (11)	
C26	−0.0083 (4)	−0.1183 (3)	0.2518 (9)	0.084 (2)	
H26	−0.0511	−0.1002	0.2995	0.101*	
C27	0.0043 (4)	−0.1848 (3)	0.2232 (10)	0.085 (2)	
H27	−0.0270	−0.2218	0.2470	0.102*	
C28	0.0737 (4)	−0.1854 (3)	0.1509 (9)	0.0819 (19)	
H28	0.0973	−0.2250	0.1173	0.098*	

### Atomic displacement parameters (Å^2^)

**Table:**

	*U*^11^	*U*^22^	*U*^33^	*U*^12^	*U*^13^	*U*^23^
N1	0.045 (2)	0.044 (2)	0.096 (3)	−0.0036 (19)	0.011 (2)	−0.006 (2)
N2	0.045 (2)	0.044 (2)	0.100 (4)	−0.0009 (18)	0.010 (2)	−0.001 (2)
N3	0.046 (2)	0.048 (3)	0.098 (3)	−0.001 (2)	0.011 (2)	0.003 (2)
N4	0.050 (2)	0.050 (3)	0.082 (3)	−0.005 (2)	0.002 (2)	0.005 (2)
N5	0.067 (3)	0.064 (4)	0.136 (5)	−0.015 (3)	0.005 (3)	0.024 (3)
N6	0.054 (2)	0.038 (2)	0.091 (3)	0.001 (2)	0.013 (2)	−0.002 (2)
N7	0.047 (2)	0.043 (3)	0.079 (3)	0.0015 (19)	0.015 (2)	−0.006 (2)
N8	0.042 (2)	0.041 (2)	0.081 (3)	−0.0020 (18)	0.014 (2)	−0.005 (2)
N9	0.050 (2)	0.040 (2)	0.067 (3)	0.0012 (19)	0.010 (2)	−0.003 (2)
N10	0.068 (3)	0.044 (3)	0.097 (4)	0.006 (2)	0.027 (3)	−0.007 (2)
C1	0.052 (3)	0.039 (3)	0.062 (3)	0.000 (2)	0.020 (2)	0.002 (2)
C2	0.074 (3)	0.042 (3)	0.063 (3)	−0.005 (3)	0.037 (3)	0.001 (2)
C3	0.099 (5)	0.046 (3)	0.079 (4)	0.013 (3)	0.043 (4)	0.000 (3)
C4	0.069 (4)	0.078 (4)	0.090 (5)	0.023 (3)	0.024 (3)	0.017 (4)
C5	0.060 (3)	0.069 (4)	0.087 (4)	0.003 (3)	0.018 (3)	0.005 (3)
C6	0.056 (3)	0.044 (3)	0.069 (3)	0.004 (2)	0.018 (2)	−0.001 (2)
C7	0.096 (4)	0.064 (4)	0.080 (4)	−0.021 (3)	0.032 (3)	−0.016 (3)
C8	0.048 (3)	0.047 (3)	0.059 (3)	−0.006 (2)	0.004 (2)	0.001 (2)
C9	0.057 (3)	0.056 (3)	0.076 (4)	0.009 (3)	0.027 (3)	0.008 (3)
C10	0.052 (3)	0.062 (3)	0.068 (4)	−0.008 (3)	0.016 (3)	0.010 (3)
C11	0.052 (3)	0.047 (3)	0.068 (3)	−0.005 (2)	0.010 (2)	0.006 (2)
C12	0.069 (4)	0.059 (4)	0.108 (5)	0.004 (3)	0.011 (3)	−0.008 (3)
C13	0.094 (5)	0.060 (4)	0.126 (6)	0.008 (4)	−0.018 (4)	0.007 (4)
C14	0.083 (5)	0.053 (4)	0.166 (8)	−0.017 (4)	−0.019 (5)	0.033 (4)
C15	0.0806 (16)	0.0625 (15)	0.112 (2)	−0.0139 (12)	0.0506 (16)	−0.0222 (14)
C16	0.0806 (16)	0.0625 (15)	0.112 (2)	−0.0139 (12)	0.0506 (16)	−0.0222 (14)
C17	0.0806 (16)	0.0625 (15)	0.112 (2)	−0.0139 (12)	0.0506 (16)	−0.0222 (14)
C18	0.0806 (16)	0.0625 (15)	0.112 (2)	−0.0139 (12)	0.0506 (16)	−0.0222 (14)
C19	0.0806 (16)	0.0625 (15)	0.112 (2)	−0.0139 (12)	0.0506 (16)	−0.0222 (14)
C20	0.0806 (16)	0.0625 (15)	0.112 (2)	−0.0139 (12)	0.0506 (16)	−0.0222 (14)
C21	0.154 (7)	0.066 (4)	0.103 (6)	0.021 (5)	0.053 (5)	0.023 (4)
C22	0.048 (3)	0.047 (3)	0.061 (3)	−0.006 (2)	0.013 (2)	0.001 (2)
C23	0.055 (3)	0.054 (3)	0.077 (4)	−0.005 (3)	0.028 (3)	0.002 (3)
C24	0.047 (3)	0.052 (3)	0.071 (3)	0.003 (2)	0.022 (2)	−0.008 (3)
C25	0.042 (2)	0.046 (3)	0.051 (3)	0.003 (2)	0.007 (2)	−0.005 (2)
C26	0.070 (4)	0.052 (4)	0.139 (6)	0.005 (3)	0.044 (4)	0.011 (3)
C27	0.079 (4)	0.043 (3)	0.136 (6)	−0.003 (3)	0.025 (4)	0.001 (3)
C28	0.077 (4)	0.046 (4)	0.123 (6)	0.008 (3)	0.017 (4)	−0.011 (3)

### Geometric parameters (Å, °)

**Table:**

N1—C8	1.366 (6)	C8—C9	1.406 (7)
N1—C1	1.426 (6)	C9—C10	1.342 (7)
N1—H1	0.8600	C9—H9	0.9300
N2—C8	1.321 (6)	C10—C11	1.383 (7)
N2—N3	1.364 (6)	C10—H10	0.9300
N3—C11	1.306 (6)	C12—C13	1.362 (8)
N4—C12	1.338 (7)	C12—H12	0.9300
N4—N5	1.366 (6)	C13—C14	1.380 (11)
N4—C11	1.406 (6)	C13—H13	0.9300
N5—C14	1.321 (9)	C14—H14	0.9300
N6—C22	1.356 (6)	C15—C16	1.3900
N6—C15	1.407 (5)	C15—C20	1.3900
N6—H6A	0.8600	C16—C17	1.3900
N7—C22	1.335 (6)	C16—C21	1.495 (8)
N7—N8	1.349 (5)	C17—C18	1.3900
N8—C25	1.306 (6)	C17—H17	0.9300
N9—C26	1.341 (7)	C18—C19	1.3900
N9—N10	1.345 (6)	C18—H18	0.9300
N9—C25	1.411 (5)	C19—C20	1.3900
N10—C28	1.310 (7)	C19—H19	0.9300
C1—C6	1.383 (7)	C20—H20	0.9300
C1—C2	1.383 (6)	C21—H21A	0.9600
C2—C3	1.412 (8)	C21—H21B	0.9600
C2—C7	1.485 (8)	C21—H21C	0.9600
C3—C4	1.367 (9)	C22—C23	1.404 (7)
C3—H3	0.9300	C23—C24	1.358 (7)
C4—C5	1.356 (8)	C23—H23	0.9300
C4—H4	0.9300	C24—C25	1.387 (7)
C5—C6	1.372 (7)	C24—H24	0.9300
C5—H5	0.9300	C26—C27	1.347 (8)
C6—H6	0.9300	C26—H26	0.9300
C7—H7A	0.9600	C27—C28	1.371 (9)
C7—H7B	0.9600	C27—H27	0.9300
C7—H7C	0.9600	C28—H28	0.9300
			
C8—N1—C1	125.3 (4)	N4—C12—C13	107.4 (6)
C8—N1—H1	117.3	N4—C12—H12	126.3
C1—N1—H1	117.3	C13—C12—H12	126.3
C8—N2—N3	119.3 (4)	C12—C13—C14	104.4 (6)
C11—N3—N2	119.5 (4)	C12—C13—H13	127.8
C12—N4—N5	112.0 (5)	C14—C13—H13	127.8
C12—N4—C11	129.1 (5)	N5—C14—C13	113.3 (6)
N5—N4—C11	118.9 (5)	N5—C14—H14	123.4
C14—N5—N4	103.1 (6)	C13—C14—H14	123.4
C22—N6—C15	123.9 (4)	C16—C15—C20	120.0
C22—N6—H6A	118.1	C16—C15—N6	120.9 (3)
C15—N6—H6A	118.1	C20—C15—N6	119.0 (3)
C22—N7—N8	119.8 (4)	C17—C16—C15	120.0
C25—N8—N7	119.7 (4)	C17—C16—C21	119.0 (4)
C26—N9—N10	111.4 (4)	C15—C16—C21	121.0 (4)
C26—N9—C25	127.8 (4)	C16—C17—C18	120.0
N10—N9—C25	120.7 (4)	C16—C17—H17	120.0
C28—N10—N9	103.7 (5)	C18—C17—H17	120.0
C6—C1—C2	120.9 (5)	C17—C18—C19	120.0
C6—C1—N1	119.5 (4)	C17—C18—H18	120.0
C2—C1—N1	119.6 (4)	C19—C18—H18	120.0
C1—C2—C3	115.5 (5)	C20—C19—C18	120.0
C1—C2—C7	121.9 (5)	C20—C19—H19	120.0
C3—C2—C7	122.6 (5)	C18—C19—H19	120.0
C4—C3—C2	122.8 (5)	C19—C20—C15	120.0
C4—C3—H3	118.6	C19—C20—H20	120.0
C2—C3—H3	118.6	C15—C20—H20	120.0
C5—C4—C3	120.5 (6)	C16—C21—H21A	109.5
C5—C4—H4	119.7	C16—C21—H21B	109.5
C3—C4—H4	119.7	H21A—C21—H21B	109.5
C4—C5—C6	118.3 (6)	C16—C21—H21C	109.5
C4—C5—H5	120.8	H21A—C21—H21C	109.5
C6—C5—H5	120.8	H21B—C21—H21C	109.5
C5—C6—C1	122.0 (5)	N7—C22—N6	115.8 (4)
C5—C6—H6	119.0	N7—C22—C23	120.9 (4)
C1—C6—H6	119.0	N6—C22—C23	123.2 (5)
C2—C7—H7A	109.5	C24—C23—C22	118.9 (5)
C2—C7—H7B	109.5	C24—C23—H23	120.5
H7A—C7—H7B	109.5	C22—C23—H23	120.5
C2—C7—H7C	109.5	C23—C24—C25	116.7 (5)
H7A—C7—H7C	109.5	C23—C24—H24	121.7
H7B—C7—H7C	109.5	C25—C24—H24	121.7
N2—C8—N1	116.6 (4)	N8—C25—C24	123.8 (4)
N2—C8—C9	121.5 (5)	N8—C25—N9	115.6 (4)
N1—C8—C9	121.8 (5)	C24—C25—N9	120.6 (4)
C10—C9—C8	118.7 (5)	N9—C26—C27	107.6 (6)
C10—C9—H9	120.7	N9—C26—H26	126.2
C8—C9—H9	120.7	C27—C26—H26	126.2
C9—C10—C11	117.3 (5)	C26—C27—C28	104.1 (6)
C9—C10—H10	121.4	C26—C27—H27	127.9
C11—C10—H10	121.4	C28—C27—H27	127.9
N3—C11—C10	123.6 (5)	N10—C28—C27	113.2 (6)
N3—C11—N4	115.2 (5)	N10—C28—H28	123.4
C10—C11—N4	121.1 (5)	C27—C28—H28	123.4
			
C8—N2—N3—C11	0.8 (8)	N4—C12—C13—C14	0.4 (8)
C12—N4—N5—C14	−0.1 (7)	N4—N5—C14—C13	0.4 (8)
C11—N4—N5—C14	178.6 (5)	C12—C13—C14—N5	−0.5 (9)
C22—N7—N8—C25	0.8 (7)	C22—N6—C15—C16	144.5 (4)
C26—N9—N10—C28	−1.0 (7)	C22—N6—C15—C20	−34.3 (6)
C25—N9—N10—C28	−176.7 (5)	C20—C15—C16—C17	0.0
C8—N1—C1—C6	−39.5 (7)	N6—C15—C16—C17	−178.8 (4)
C8—N1—C1—C2	140.1 (5)	C20—C15—C16—C21	−179.7 (5)
C6—C1—C2—C3	−0.3 (7)	N6—C15—C16—C21	1.5 (5)
N1—C1—C2—C3	−179.9 (4)	C15—C16—C17—C18	0.0
C6—C1—C2—C7	−179.6 (5)	C21—C16—C17—C18	179.7 (5)
N1—C1—C2—C7	0.8 (7)	C16—C17—C18—C19	0.0
C1—C2—C3—C4	1.2 (8)	C17—C18—C19—C20	0.0
C7—C2—C3—C4	−179.5 (6)	C18—C19—C20—C15	0.0
C2—C3—C4—C5	−1.2 (9)	C16—C15—C20—C19	0.0
C3—C4—C5—C6	0.3 (9)	N6—C15—C20—C19	178.8 (4)
C4—C5—C6—C1	0.6 (8)	N8—N7—C22—N6	179.7 (4)
C2—C1—C6—C5	−0.6 (8)	N8—N7—C22—C23	2.7 (7)
N1—C1—C6—C5	179.0 (5)	C15—N6—C22—N7	157.2 (4)
N3—N2—C8—N1	178.9 (4)	C15—N6—C22—C23	−25.8 (8)
N3—N2—C8—C9	1.8 (8)	N7—C22—C23—C24	−3.9 (8)
C1—N1—C8—N2	162.4 (5)	N6—C22—C23—C24	179.3 (5)
C1—N1—C8—C9	−20.5 (8)	C22—C23—C24—C25	1.6 (8)
N2—C8—C9—C10	−4.1 (8)	N7—N8—C25—C24	−3.1 (8)
N1—C8—C9—C10	179.0 (5)	N7—N8—C25—N9	176.5 (4)
C8—C9—C10—C11	3.6 (8)	C23—C24—C25—N8	1.8 (8)
N2—N3—C11—C10	−1.2 (8)	C23—C24—C25—N9	−177.7 (5)
N2—N3—C11—N4	177.1 (5)	C26—N9—C25—N8	−5.7 (8)
C9—C10—C11—N3	−1.2 (8)	N10—N9—C25—N8	169.3 (5)
C9—C10—C11—N4	−179.3 (5)	C26—N9—C25—C24	173.9 (6)
C12—N4—C11—N3	−6.2 (9)	N10—N9—C25—C24	−11.1 (7)
N5—N4—C11—N3	175.3 (5)	N10—N9—C26—C27	1.0 (8)
C12—N4—C11—C10	172.1 (6)	C25—N9—C26—C27	176.3 (5)
N5—N4—C11—C10	−6.3 (8)	N9—C26—C27—C28	−0.5 (8)
N5—N4—C12—C13	−0.2 (8)	N9—N10—C28—C27	0.6 (7)
C11—N4—C12—C13	−178.7 (6)	C26—C27—C28—N10	−0.1 (8)

### Hydrogen-bond geometry (Å, °)

**Table:**

*D*—H···*A*	*D*—H	H···*A*	*D*···*A*	*D*—H···*A*
N1—H1···N7^i^	0.86	2.26	3.086 (5)	162
N6—H6A···N2^ii^	0.86	2.26	3.065 (6)	156
C7—H7C···N8^i^	0.96	2.53	3.482 (8)	175
C21—H21C···N3^ii^	0.96	2.59	3.519 (10)	163
C6—H6···Cg1	0.93	2.80	3.529 (6)	136

Symmetry codes: (i) *x*+1/2, −*y*+1/2, *z*+1/2; (ii) *x*−1/2, −*y*+1/2, *z*−1/2.
